# The Drowning Season

**Published:** 1884-11

**Authors:** 


### THE DROWNING SEASON.

IT must, we presume, be accepted as a general proposition capable of particular application in the most vexatious directions and specialties that any and every sport, exercise, and pastime which involves a certain amount of personal risk will have an inevitable attraction for English folk of all classes and ages. On no other principle is it possible to account for the curious fact that, although year after year many lives are ruthlessly and uselessly sacrificed in such practices as bathing, boating, hunting, and skating, no lover of these several pursuits is deterred from indulging in them by the peril incurred. On the contrary, it is beyond question that this very peril adds zest to the pastimes. When, therefore, we encounter a paragraph in the press under the above ominous heading, giving particulars of eleven deaths by what are, almost facetiously, termed "accidents" in the course of a few days, we do not dwell long on the mournful epitome, but pass it over with something of the same feeling as may be experienced on reading the bare statistical statement of a death-rate. Nevertheless, it is a grim fact that every one of these lives might have been saved by a little timely precaution. Either the bath was taken in too deep water or at an unsuitable spot, or there was some neglect of obvious measures and conditions of safety. Nothing would be gained by reiterating the warnings we have repeatedly given as to sudden plunges into cold water if the heart be weak, or there be a tendency to cramp; as to severe trials of strenghth in buffeting with heavy waves or trying to swim long distances; and as to going into the water weak or exhausted by want of food, or with a stomach so loaded that disturbances of the circulatory and nervous systems must almost inevitably ensue if there be even a moderate amount of congestion set up by the immersion in a fluid colder than the atmosphere. Tell an English man or boy that any thing is dangerous, and he will burn to do it. If he be weak, he will try to behave as though he were strong. If he be unfit, by reason of physical disability of some sort, for a particular exercise, he will yearn, above all things, to do the impossible, and to hazard the extremely perilous. In health he will scorn safe-guards and precautions as signs and tokens of cowardice. We do not think that mere carelessness is so common as some people are apt to suppose. We believe it is downright foolhardiness that leads to "accidents" of the class represented as "deaths by drowning." —*London Lancet.*

